# Supplementary material for: Chito-Oligosaccharide and Propolis Extract of Stingless Bees Reduce the Infection Load of Nosema ceranae in Apis dorsata (Hymenoptera: Apidae)
Source: J Fungi (Basel). 2022 Dec 22;9(1):20. doi: 10.3390/jof9010020 (PMC9861836; doi:10.3390/jof9010020)
Supplement: Supplementary file 1 [file jof-09-00020-s001.zip › jof-2105683-supplementary.pdf]

Journal of *Fungi*

Supplementary materials

Molecular identification

Midguts of *Apis florea*, *A. mellifera,* and *A. dorsata* were removed (n= 20) and transferred to 1.5 mL microcentrifuge tubes containing 200 µL distilled water, these were homogenized using a homogenizer that fit tightly into each tube. Each tube was centrifuged at 6,000 g for 10 min to separate *Nosema* spores from bee pollen and other adhering tissues. The top white sediment was collected and transferred into another 1.5 mL microcentrifuge tube containing 100 µL of distilled water. This was then repeated two times until pure *Nosema* spores (with any adhering pollens) became visible. Spores were kept frozen at -20° C until further analysis.

DNA extraction from spore isolate

DNA was extracted from homogenates by using the Chelex method (Walsh et al., 1991). Firstly, approximately 10 µg of glass beads was added into 50 µL of spore isolate solution, then it was placed on cell disrupter at max speed (5,800 g) for 5 min. We then centrifuged this for 1 min at max speed (12,000 g). A 100 µL Chelex solution was added into each well of a PCR plate before adding 50 µL of disrupted spores and 5 µL of proteinase K. The plate was then placed in thermocycler ("Chelex[1]" program) and centrifuged again (2 min at 12,000 g). A 100 µL of the supernatant was transferred into a new plate and then stored at -20° C until further analysis

Multiplex PCR

For the multiplex PCR amplification of partial 16S rRNA (=SSU rRNA) gene fragments, the primer combinations can be found in Table S1. Primers were designed based on the alignment of all available sequence data in GenBank of 16S rRNA gene from *N. apis* and *N. ceranae*.

Table S1. List of primer sets for the detection of *N. ceranae* by PCR.

Name	Type	Primer sequence
Mnceranae-F	forward primer	5'-CGT-TAA-AGT-GTA-GAT-AAG-ATG-TT-3'
Mnapis-F	forward primer	5'-GCA-TGT-CTT-TGA-CGT-ACT-ATG-3'
Muniv-R	reverse primer	5'-GAC-TTA-GTA-GCC-GTC-TCT-C-3'

Note: PCR product size: for *N. ceranae*: 143 bp; for *N. apis*: 224 bp.

PCR conditions

The following PCR reaction mixture was used: 1.0 µL of DNA (ca. 1.0 ng); 0.05 µL of Taq polymerase; 1 µL buffer; 0.2 µL dNTPs; 0.4 µL of Mnceranae-F; 0.4 µL of Mnapis-F; 0.5 µL of Muniv-R; ddH2O for a final volume of 10 µL. Amplification was carried out in a thermocycler under the following conditions: an initial denaturation step of 95° C for 2 min , 35 cycles of 95° C for 30 s, 55° C for 30 s and 72° C for 60 s, with a final extension step of 72° C for 5 min.

Visualization

The amplified products were separated using a 2% agarose gel. The molecular size of PCR products was determined by staining them with SYBR green, and visualizing them using an UviTec gel documentation system.

Molecular identification of *Nosema* species

The PCR products of 60 samples (20 samples of each honey bee species) revealed that only *N. ceranae* was present, with a product length of 145-147 bp (Fig. S1).

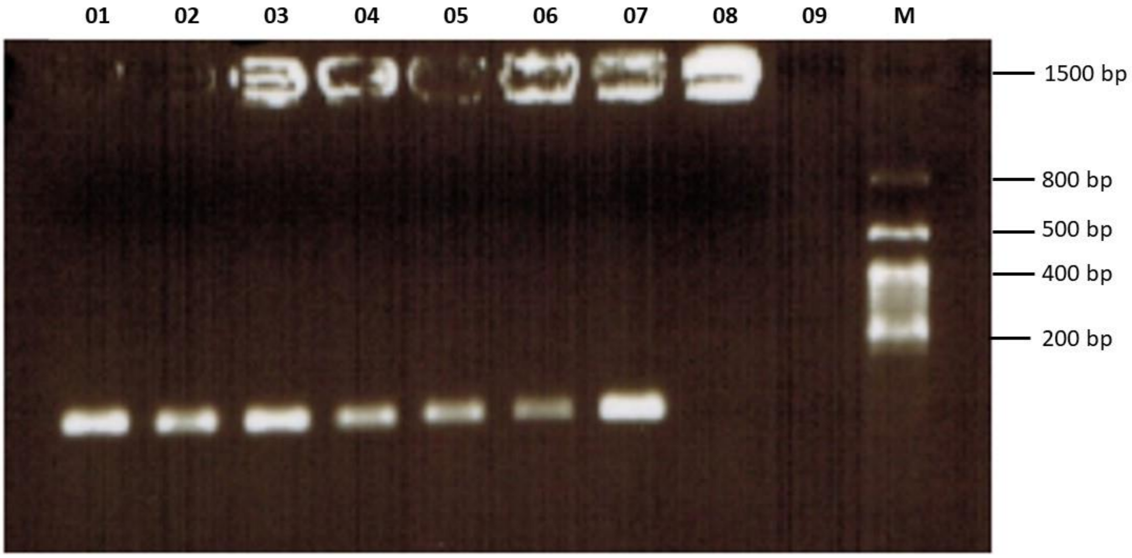

Figure S1. A 2% Agarose gel electrophoresis of PCR amplified products for 16S rRNA gene; *N. ceranae* 143 bp; Lane M: l00bp ladder, 01-NAF1, 02-NAF2, 03-NAF3, 04-NAD4, 05- NAD5, 06-NAM6, 07-positive for *N. ceranae*, 08-negative, 09-H_2O (NAD = *Nosema* spores from *A. dorsata* after fed with *N. ceranae* from *A. mellifera*, NAF = *Nosema* spores extracted from *A. florea*, NAM = *Nosema* spores extracted from propagated bees, *A. mellifera* (after inoculated with *Nosema* spores from *A. florea* for 14 days).

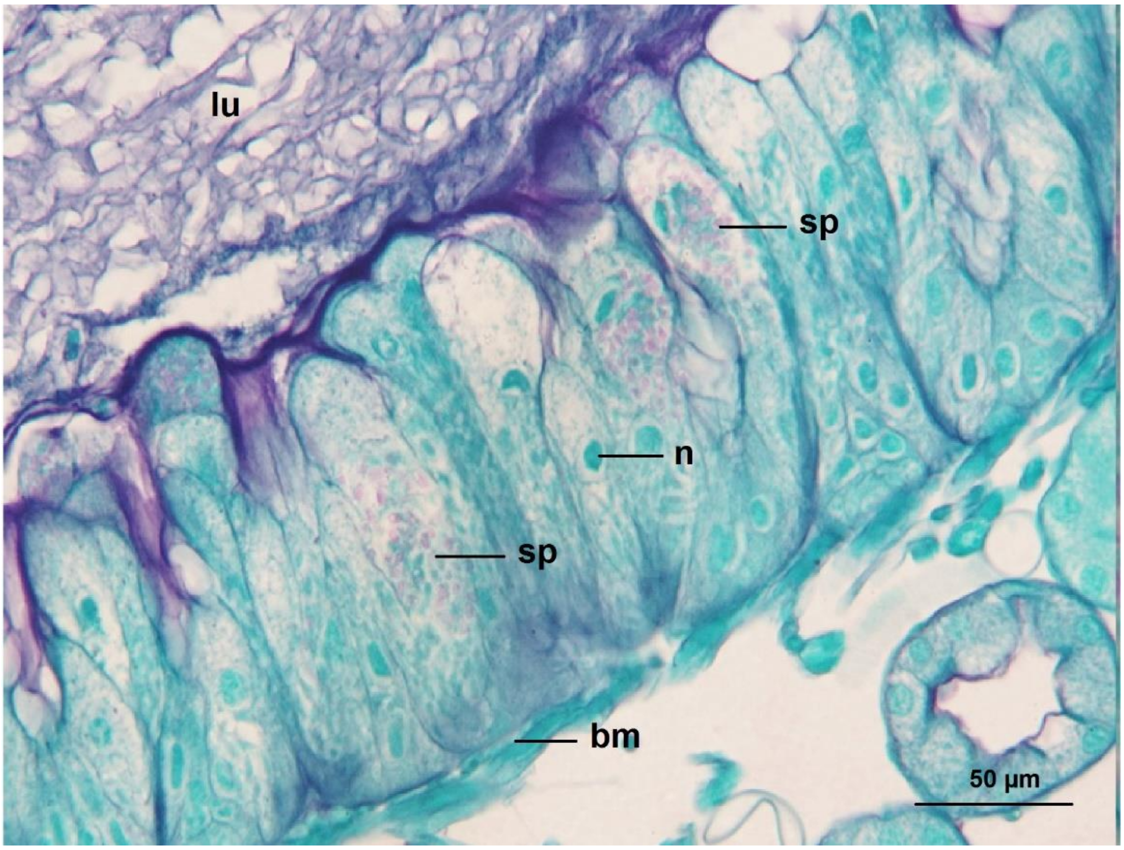

Figure S2. A light micrograph of *A. dorsata* ventricular cells, is from a NO-50P bee on day 14 p.i. (PAS, 400x). *Nosema* spores are stained pink with PAS. Abbreviations: bm, basement membrane; lu, lumen of the midgut; mg, Malpighian tubule; n, nucleus of ventricular cell; sp, *N. ceranae* spores.

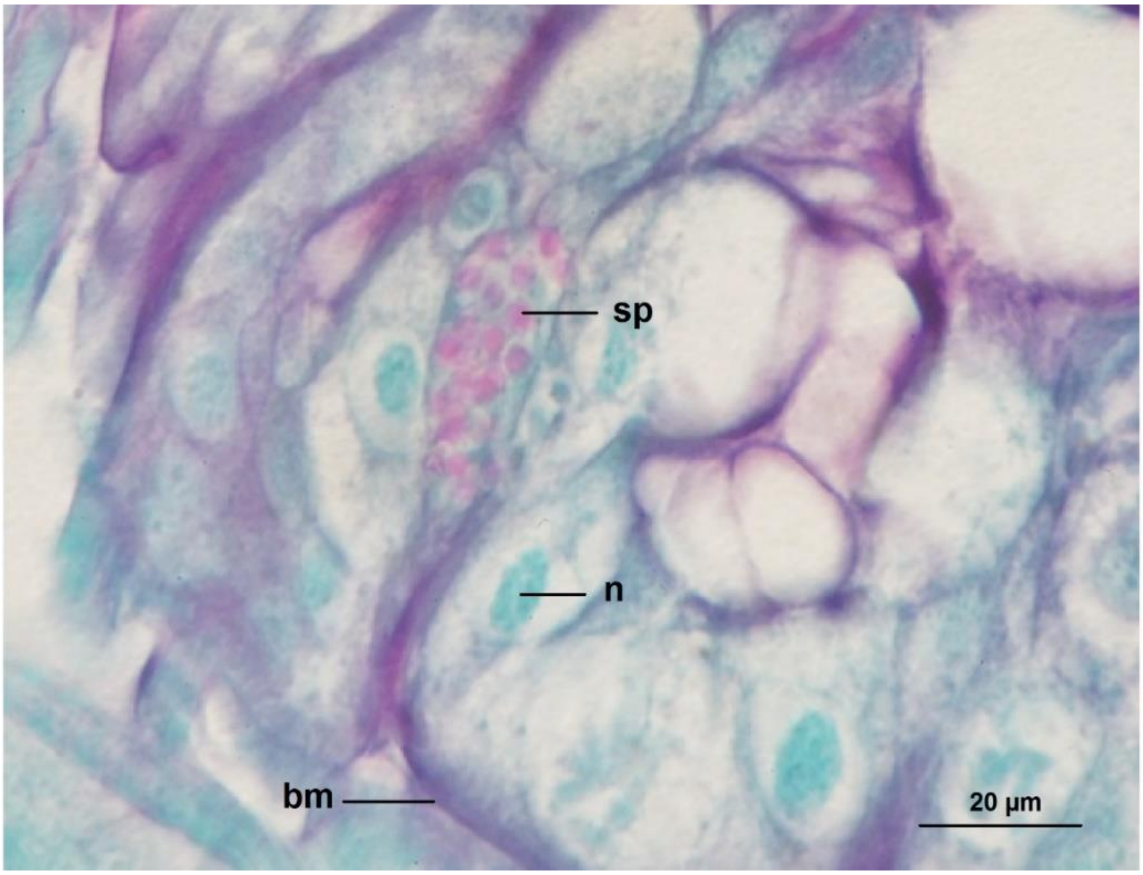

Figure S3. A light micrograph of *A. dorsata* ventricular cells, is from a NO-0.5COS bee (PAS, 1,000x). *Nosema* spores are stained pink with PAS. Abbreviations: bm, basement membrane; n, nucleus of ventricular cell; sp, *N. ceranae* spores.
